# Long-term outcomes of patients with end-stage kidney disease due to membranoproliferative glomerulonephritis: an ANZDATA registry study

**DOI:** 10.1186/s12882-019-1605-6

**Published:** 2019-11-21

**Authors:** Gregory J. Wilson, Yeoungjee Cho, Armando Teixiera-Pinto, Nicole Isbel, Scott Campbell, Carmel Hawley, David W. Johnson

**Affiliations:** 1 Department of Nephrology, Princess Alexandra Hospital, 199 Ipswich Rd, Wooloongabba, Brisbane, Australia; 2 Australasian Kidney Trials Network, University of Queensland, Brisbane, Australia; 3 The University of Sydney, Sydney, Australia; 4 Translational Research Institute, Brisbane, Australia

**Keywords:** Dialysis, End-stage kidney disease, Kidney transplantation, Membranoproliferative glomerulonephritis, Mesangiocapillary glomerulonephritis

## Abstract

**Background:**

Membranoproliferative glomerulonephritis (MPGN) is an uncommon cause of end stage kidney disease (ESKD) and the clinical outcomes of patients with MPGN who commence kidney replacement therapy have not been comprehensively studied.

**Methods:**

All adult patients with ESKD due to glomerulonephritis commencing kidney replacement therapy in Australia and New Zealand from January 1, 1996 to December 31, 2016 were reviewed. Patients with ESKD due to MPGN were compared to patients with other forms of glomerulonephritis. Patient survival on dialysis and following kidney transplantation, kidney recovery on dialysis, time to transplantation, allograft survival, death-censored allograft survival and disease recurrence post-transplant were compared between the two groups using Kaplan Meier survival curves and Cox proportional hazards regression.

**Results:**

Of 56,481 patients included, 456 (0.8%) had MPGN and 12,660 (22.4%) had another form of glomerulonephritis. Five-year patient survival on dialysis and following kidney transplantation were similar between patients with ESKD from MPGN and other forms of glomerulonephritis (Dialysis: 59% vs. 62% *p* = 0.61; Transplant: 93% vs. 93%, *p* = 0.49). Compared to patients with other forms of glomerulonephritis, patients with MPGN had significantly poorer 5-year allograft survival (70% vs. 81% respectively, *p* = 0.02) and death censored allograft survival (74% vs. 87%, respectively; *p* < 0.01). The risk of disease recurrence was significantly higher in patients with MPGN compared to patients with other glomerulonephritidites (18% vs. 5%; *p* < 0.01). In patients with MPGN who had allograft loss, patients with MPGN recurrence had a significantly shorter time to allograft loss compared to patients with MPGN who had allograft loss due to any other cause (median time to allograft loss 3.2 years vs. 4.4 years, *p* < 0.01).

**Conclusions:**

Compared with other forms of glomerulonephritis, patients with MPGN experienced comparable rates of survival on dialysis and following kidney transplantation, but significantly higher rates of allograft loss due to disease recurrence.

## Background

Membranoproliferative (mesangiocapillary) glomerulonephritis (MPGN) is a rare cause of end stage kidney disease (ESKD) [1]. The reported incidence of ESKD due to MPGN is 0.33% [1] and has been decreasing over the last two decades [2]. MPGN is defined histologically and characterized by diffuse proliferative lesions, mesangial hypercellularity and endocapillary proliferation with double contour formation [3–5]. MPGN can be idiopathic but is more commonly secondary to systemic inflammatory conditions [1, 3] including chronic infections (most commonly hepatitis C infection), autoimmune diseases, plasma cell dyscrasias and cryoglobulinemia [1, 6]. Traditionally, MPGN has been categorized as type 1, 2 or 3 based on the appearance of immune deposits under observed electron microscopy [7]. However, recent advances in the understanding of the pathophysiology of MPGN have resulted in the reclassification of MPGN into two entities based on the mechanism of immune injury to the glomerulus: C3 glomerulonephritis (C3GN) and immune complex MPGN (ICGN) [7].

MPGN is known to be a progressive disease with limited treatment options and over 50% of patients with MPGN progress to ESKD within 10 years and require kidney replacement therapy (KRT) [1, 8]. Previous studies of clinical outcomes in patients with MPGN receiving KRT have been confined to kidney transplant recipients and have demonstrated significantly worse allograft outcomes, including higher rates of graft loss compared to other ESKD patients [1, 3, 6, 9–11]. This has been attributed to an increased risk of disease recurrence in MPGN compared to other forms of glomerulonephritis (GN) [1, 11]. However, these studies have largely been limited to earlier transplant eras, such that outcomes in the setting of contemporary immunosuppressive regimens and transplant care are uncertain. Furthermore, whether the outcomes of patients with MPGN receiving chronic dialysis are worse than those of patients suffering from other types of GN remains unknown. Given that not all patients will proceed to kidney transplantation, it is crucial to determine whether chronic dialysis patients with MPGN are also susceptible to poorer outcomes.

The present study sought to examine potential risks specific to MPGN patients undergoing KRT in a contemporary setting, using data from the Australia and New Zealand Dialysis and Transplantation (ANZDATA) registry.

## Methods

### Study population

Data were obtained from patients enrolled in the ANZDATA registry. All patients with ESKD from January 1, 1996 to December 31, 2016 in Australia and New Zealand were included (Additional file 1: Figure S1). Patients with ESKD caused by glomerulonephritis were selected from this group. These patients were then categorized into two cohorts: patients with ESKD caused by MPGN and patients with ESKD caused by any other form of glomerulonephritis (all other GN). Only patients with a diagnosis of a primary GN were included in the all other GN group. Data on MPGN subtype was not available. Patients with missing demographic or co-morbidity data were excluded from the study. These patients represented less than 1% of the selected patient cohort.

### Data collection

Baseline characteristics were evaluated at the initiation of dialysis or, if pre-emptively transplanted, at the time of transplantation. They included age, sex, ethnicity, smoking status, hepatitis C antibody status, diabetes mellitus, chronic lung disease, coronary artery disease, peripheral vascular disease, cerebrovascular disease and any previous diagnosis of cancer. Dialysis and transplant details were also obtained including the commencement date of dialysis, date of transplantation, date of kidney function recovery if applicable, and whether the patient was a late referral for KRT (defined as referral to a nephrologist within 3 months of KRT commencement). Patients were categorized into two dialysis or transplant eras: from 1996 to 2006 or 2007–2016, based on the date of commencement of dialysis or transplantation.

### Clinical outcomes

The primary outcome was patient survival. Secondary outcomes evaluated included cause of death, kidney function recovery on dialysis, time to transplantation, allograft survival, cause of allograft loss and disease recurrence post-transplant.

### Statistical analyses

Categorical variables were expressed as numbers and percentages and continuous variables as mean with standard deviations or median with interquartile range based on distribution. Differences between MPGN and all other GN groups were assessed with Chi squared or Fisher’s exact test for categorical variables, and students t-test for continuous variables.

Propensity score matching was utilized to compare primary and secondary outcomes between MPGN and all other GN patients for both dialysis and transplantation cohorts using the MatchIt software package [12]. Propensity scores were calculated using a logistic regression model for the likelihood of developing MPGN using patient age, sex, ethnicity, all available co-morbidities, late referral for KRT, and dialysis or transplantation era. A 1:1 nearest-neighbor matching algorithm was used with no replacement. A maximum matching caliper distance of 0.25 standard deviations of the logit of the propensity score was permitted and, if no match was possible, the patient was excluded from matching. Standardized mean differences were calculated for each variable used in matching.

Patient survival on dialysis, patient survival following kidney transplantation, kidney function recovery on dialysis, kidney allograft survival and death censored kidney allograft survival were assessed by Kaplan Meier analysis, multivariable Cox proportional hazards regression and competing risk analysis (Fine and Gray method) [13] using the survival and survminer packages [14, 15]. For patients with multiple kidney transplants, outcomes following their first transplant only were considered. Covariates included in the Cox models were age, sex, ethnicity, dialysis or transplantation era, hepatitis C antibody status, smoking status, presence of diabetes, coronary artery disease, peripheral vascular disease, cerebrovascular disease, previous diagnosis of cancer, KRT type and late referral for KRT. Schoenfeld residuals were used to assess proportional hazards assumptions. Patient survival on dialysis was censored for kidney function recovery, loss to follow-up, kidney transplantation and end of study. Kidney function recovery was censored for death, loss to follow-up, kidney transplantation and end of study. Patient survival following kidney transplantation was censored for allograft failure, loss to follow-up and end of study. Death-censored kidney allograft survival was censored for death, loss to follow-up and end of study. Competing-risk for patients with and without MPGN were calculated. Kidney transplantation was a competing risk for death on dialysis, patient death was the competing risk for death censored allograft failure, and allograft failure was the competing risk for death following kidney transplantation. Data processing and analysis was performed using R [16]. *P* values less than 0.05 were deemed statistically significant.

## Results

### Population characteristics

Between 1996 and 2016, a total of 56,481 patients received dialysis in Australia and New Zealand; 13,462 of these received a kidney transplant. Within this group, 456 (0.8%) patients had ESKD secondary to MPGN (441 requiring dialysis and 15 with pre-emptive transplants) and 12,660 (22.4%) had any other form of glomerulonephritis as the cause of their ESKD (all other GN) (Table 1). All patients with MPGN had a renal biopsy confirming their diagnosis while 74.6% of patients with all other GN were biopsy confirmed. The most common GN in patients with any other GN was IgA nephropathy (3196, 27%; Additional file 1: Table S1). Compared with all other GN patients, those with ESKD from MPGN were more likely to be Maori or Pacific Islander (15% vs. 7%) or have a positive Hepatitis C antibody (10% vs. 1%). There was no difference in the incidence of a positive Hepatitis C antibody between different ethnicities. The incidence of MPGN as a cause of ESKD became less frequent over the two dialysis eras, with 274 patients (1.1% of all patients on KRT) developing ESKD due to MPGN between 1996 and 2006 compared to 182 (0.6%) between 2007 and 2016. The prevalence of Hepatitis C antibody positive MPGN patients remained similar across the two dialysis eras (10.2% in 96–06 vs. 10.4% in 07–16). The ethnicity of patients with MPGN also did not differ between the two dialysis eras (Additional file 1: Table S2). Rates of cancer were higher in patients with MPGN compared to those with GN (10.0% vs 7.3%).

**Table 1:** Characteristics of patients with ESKD secondary to MPGN or all other GN who commenced dialysis in Australia and New Zealand 1996-2016 (unmatched and propensity score matched patients)

Characteristic	Unmatched Cohort		Matched Cohort	
	MPGN (*n* = 441)	All other GN (*n* = 12,029)	MPGN (*n* = 440)	All other GN (*n* = 440)
Age (years)	51 (38–64)	55 (41–67)	53 (41–65)	51 (38–64)
Male	271 (61%)	7655 (64%)	271 (62%)	290 (66%)
Race				
Caucasian	299 (68%)	8948 (74%)	299 (68%)	299 (68%)
ATSI	40 (9%)	602 (5%)	40 (9%)	52 (12%)
MPI	69 (16%)	886 (7%)	68 (15%)	55 (12%)
Asian	24 (5%)	1336 (11%)	24 (5%)	27 (6%)
Other	9 (2%)	257 (2%)	9 (2%)	7 (2%)
Dialysis Era				
1996–2006	264 (60%)	6275 (52%)	263 (60%)	267 (61%)
2007–2016	177 (40%)	5754 (48%)	177 (40%)	173 (39%)
Hepatitis C Antibody Positive	47 (11%)	183 (2%)	46 (10%)	50 (11%)
Current Smoker	90 (20%)	1642 (14%)	90 (20%)	96 (22%)
Diabetes Mellitus	75 (17%)	1579 (13%)	74 (17%)	80 (18%)
Chronic Lung Disease	46 (10%)	1362 (11%)	46 (10%)	49 (11%)
Coronary Artery Disease	76 (17%)	2288 (19%)	76 (17%)	86 (20%)
Peripheral Vascular Disease	28 (6%)	872 (7%)	28 (6%)	37 (8%)
Cerebrovascular Disease	26 (6%)	812 (7%)	26 (6%)	33 (8%)
Previous history of Cancer	44 (10%)	879 (7%)	44 (10%)	40 (9%)
Late referral for KRT	97 (22%)	2798 (23%)	96 (22%)	102 (23%)
First KRT Modality				
HD	329 (75%)	8620 (72%)	329 (75%)	328 (75%)
PD	112 (25%)	3409 (28%)	111 (25%)	112 (25%)

*ATSI* Aboriginal and Torres Strait Islander, *HD* Hemodialysis, *KRT* Kidney replacement therapy, *MPI* Maori and Pacific islander, *PD* Peritoneal dialysis

The median follow up for all patients in the study was 2.74 years (IQR 0.68–4.8). For patients in the first dialysis era (1996–2006) it was 3.28 years (IQR 0.78–5.78) and for the second era (2007–2016) it was 2.31 years (IQR 0.63–3.99). Over the study period, 220 patients on dialysis with MPGN (50%) and 5234 patients with all other GN (44%) on dialysis died. The most common cause of death in both groups was cardiovascular disease (32% MPGN vs. 33% all other GN; Additional file 1: Table S3). In patients who underwent kidney transplantation, 37 patients with MPGN (19%) and 761 patients with all other GN died (14%; Additional file 1: Table S4). The most common cause of death in patients with MPGN was infection (32%) and in patients with all other GN cancer (26%).

The proportion of patients who received a kidney transplant was comparable amongst patients with MPGN (41.7%) and all other GN (43.6%; Table 2). Patients with MPGN who received a kidney transplant were more likely to be Maori or Pacific Islander (11% vs. 5%, respectively), and to have a positive Hepatitis C antibody (8% vs. 1%, respectively; Table 2).

**Table 2:** Characteristics of kidney transplant patients with ESKD secondary to MPGN and all other glomerulonephritis (unmatched and propensity score matched patients)

Characteristic	Unmatched Cohort (n)		Matched Cohort (n)	
	MPGN (*n* = 190)	All other GN (*n* = 5519)	MPGN (*n* = 188)	All other GN (*n* = 188)
Age (years)	42 (29–51)	44 (32–54)	42 (31–53)	44 (33–55)
Male	113 (59%)	3613 (65%)	111 (59%)	111 (59%)
Ethnicity				
Caucasian	147 (77%)	4228 (77%)	145 (77%)	149 (79%)
ATSI	6 (3%)	145 (3%)	6 (3%)	3 (2%)
MPI	21 (11%)	256 (5%)	21 (11%)	20 (11%)
Asian	13 (7%)	771 (14%)	13 (7%)	12 (6%)
Other	3 (2%)	119 (2%)	3 (2%)	4 (2%)
Transplant Era				
96–06	97 (51%)	2252 (41%)	97 (52%)	96 (51%)
07–16	93 (49%)	3267 (59%)	91 (48%)	92 (49%)
Hepatitis C Antibody Positive	15 (8%)	50 (1%)	13 (7%)	12 (6%)
Current Smoker	29 (15%)	572 (10%)	28 (15%)	31 (16%)
Diabetes Mellitus	14 (7%)	267 (5%)	12 (6%)	8 (4%)
Chronic Lung Disease	7 (4%)	227 (4%)	7 (4%)	8 (4%)
Coronary Artery Disease	9 (5%)	338 (6%)	9 (5%)	11 (6%)
Peripheral Vascular Disease	3 (2%)	93 (2%)	3 (2%)	4 (2%)
Cerebrovascular Disease	3 (2%)	106 (2%)	3 (2%)	6 (3%)
Cancer Diagnosis Ever	7 (4%)	161 (3%)	7 (4%)	9 (5%)
First RRT Modality				
HD	127(67%)	3435 (62%)	125 (66%)	125 (66%)
PD	48 (25%)	1453 (26%)	48 (26%)	50 (27%)
Pre-emptive Transplant	15 (8%)	631 (11%)	15 (8%)	13 (7%)

*ATSI* Aboriginal and Torres Strait Islander, *HD* Hemodialysis, *KRT* Kidney replacement therapy, *MPI* Maori and Pacific islander, *PD* Peritoneal dialysis

### Kidney function recovery

Rates of kidney function recovery after commencing dialysis were similar between the two groups (3.2% for MPGN vs 2.3% for all other GN, *p* = 0.27). Using multivariable Cox proportional hazards model analysis, there was also no difference in recovery rates for patients with MPGN compared to patients with all other GN (Hazard Ratio [HR] 1.36, 95% confidence interval [CI] 0.79–2.35, *p* = 0.26, Additional file 1: Table S5).

### Transplantation

Median duration from dialysis initiation to kidney transplantation was similar between the two groups (3.0 years for MPGN vs. 2.9 years for all other GN, *p* = 0.28; Additional file 1: Figure S4). Compared with kidney transplant patients with all other GN as their primary kidney disease, those with MPGN had higher rates of biopsy proven disease recurrence (18% vs. 5%, *p* = < 0.01). Recurrence rates were not significantly different across various ethnicities or for hepatitis C antibody status (Additional file 1: Tables S6 and S7). Rates of recurrence were similar for living vs. deceased donor (Additional file 1: Table S8). Rate of recurrence in patients with MPGN who received a pre-emptive kidney transplant recipients did not differ significantly (13%) compared to those who received a transplant once on dialysis (18%, *p* = 0.89).

**Fig. 1:**
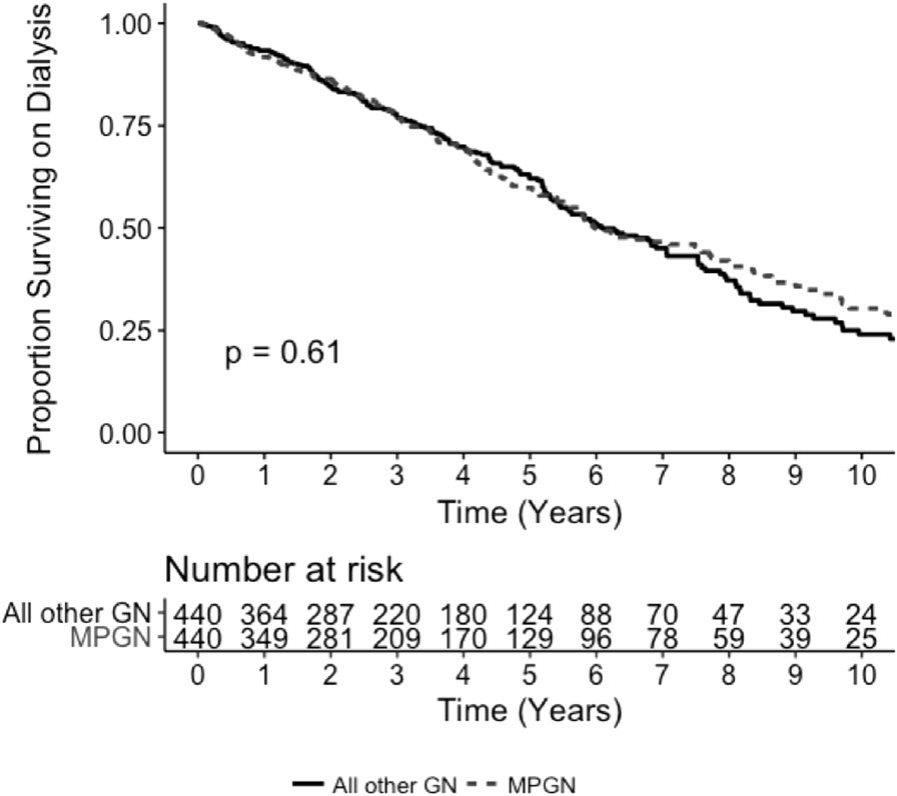
Kaplan Meier Survival analysis of kidney allograft survival in individuals with allograft loss due to MPGN recurrence vs. any other cause of allograft loss in Australia and New Zealand between 1996 and 2016

Recurrence was the second most common cause of kidney allograft loss in patients with MPGN (32%) and the sixth most common cause in patients with all other GN (6%; Additional file 1: Table S9). In patients with MPGN who had allograft loss, the median time to allograft loss was significantly shorter in patients with MPGN recurrence (3.2 years, IQR 2.1–4.3 years) compared to patients with any other cause of allograft loss (4.4 years, IQR 1.8–7.0, *p* < 0.01). Five-year allograft survival rates were also significantly lower in patients with MPGN who had allograft loss due to disease recurrence compared to MPGN patients with allograft loss from any other cause (5% vs. 43%, *p* < 0.01; Fig. 1).

### Matched cohort analysis

For each patient with MPGN, one patient with ESKD from all other GN was matched using PS matching for both dialysis and transplant patients. The characteristics of these matched cohorts were well balanced (standardized mean difference < 0.1 for all variables used for matching; Tables 1 and 2; Additional file 1: Tables S10 and S11, Figures S2 and S3). Patient survival on dialysis, following kidney transplantation, allograft survival and death censored allograft survival were analyzed in the matched cohort only.

### Patient survival on dialysis

**Fig. 2:**
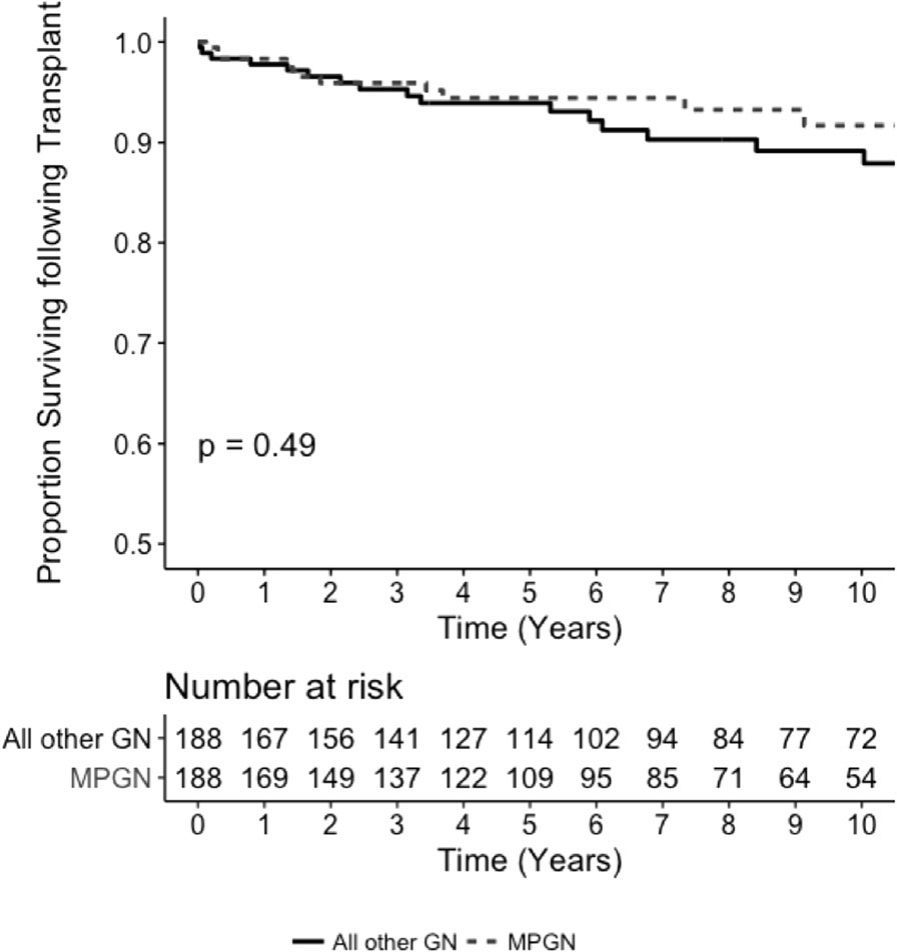
Kaplan Meier analysis of dialysis patient survival in individuals with MPGN vs. matched controls with all other GN who started dialysis in Australia and New Zealand between 1996 and 2016

Five-year survival on dialysis was 59% for patients with MPGN compared with 62% for patients with all other GN (*p* = 0.61; Fig. 2). Using multivariable Cox proportional hazards model analysis, patient survival on dialysis was not significantly different between patients with MPGN and those with all other GN (HR 1.03, 95% CI 0.85–1.27, *p* = 0.80; Table 3). Similar results were observed using competing risk regression analysis (Sub Hazard Ratio [SHR] 1.03, 95% CI 0.83 vs 1.17, *p* = 0.87; Additional file 1: Table S12).

**Table 3:** Multivariable Cox Proportional Hazards for patient mortality on dialysis

	HR (95% CI)	*P*
MPGN	1.03 (0.83–1.27)	0.80
Age	1.04 (1.03–1.05)	< 0.01
Male	0.93 (0.74–1.17)	0.53
Ethnicity		
Caucasian	Ref	< 0.01
ATSI	1.47 (1.04–2.09)	
MPI	1.77 (1.26–2.48)	
Asian	0.64 (0.35–1.19)	
Other	0.22 (0.03–1.60)	
Hepatitis C Antibody Positive	1.20 (0.83–1.73)	0.33
Current Smoker	1.43 (1.08–1.88)	0.01
Diabetes Mellitus	1.22 (0.95–1.57)	0.12
Coronary Artery Disease	1.38 (1.07–1.77)	0.01
Peripheral Vascular Disease	1.26 (0.89–1.76)	0.19
Cerebrovascular Disease	2.20 (1.55–3.11)	< 0.01
Previous Diagnosis of Cancer	1.05 (0.77–1.43)	0.77
Chronic Lung Disease	1.03 (0.77–1.37)	0.86
First KRT modality		
HD	Ref	0.57
PD	1.08 (0.84–1.39)	
Late referral to dialysis	1.09 (0.85–1.40)	0.49
Dialysis era 2007–16	0.78 (0.61–0.99)	0.04

### Patient survival following kidney transplantation

**Fig. 3:**
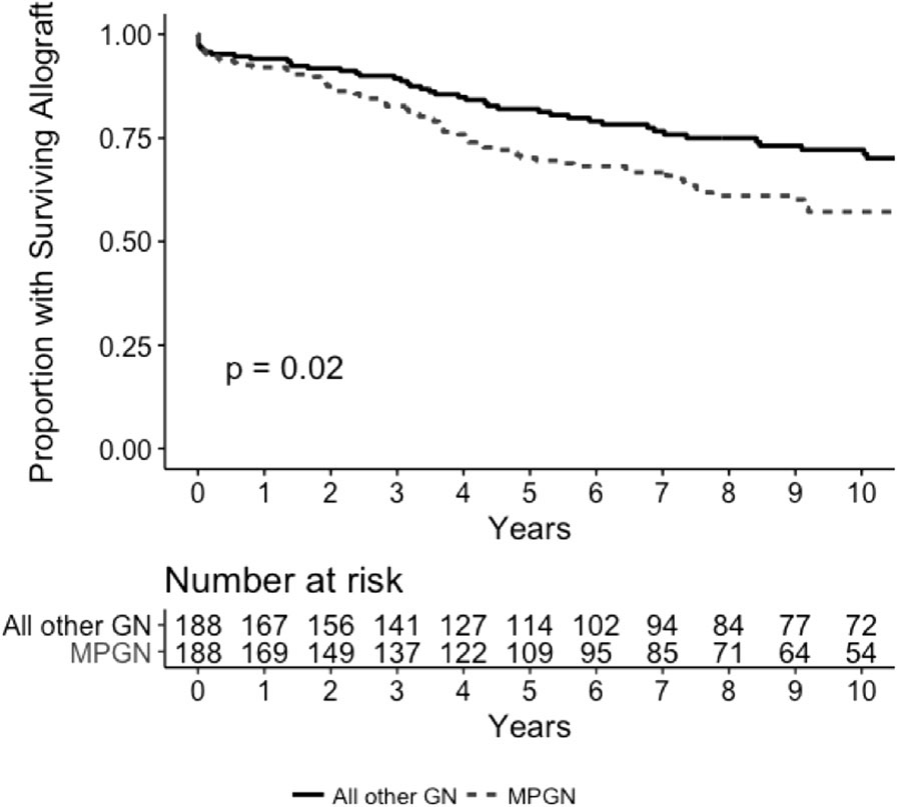
Kaplan Meier survival analysis of patient survival in individuals with MPGN vs. matched controls with all other GN who received a kidney transplant in Australia and New Zealand between 1996 and 2016

Five-year patient survival following kidney transplantation was 93% in both MPGN and all other GN patients (*p* = 0.49; Fig. 3). Using multivariable Cox proportional hazards model analysis, patient survival was not significantly different between patients with MPGN and those with all other GN (HR 0.61, 95% CI 0.29–1.26, *p* = 0.18; Table 4). Similar findings were observed with competing risks analysis (SHR 0.61, 95% CI 0.33–1.1, *p* = 0.17; Additional file 1: Table S13).

**Table 4:** Multivariable Cox Proportional Hazards for transplant patient survival

	HR (95% CI)	*P*
MPGN	0.61 (0.29–1.26)	0.18
Age	1.08 (1.04–1.12)	< 0.01
Male	0.80 (0.38–1.70)	0.57
Ethnicity		
Caucasian	Ref	0.11
ATSI	18.36 (3.22–104.72)	
MPI	2.54 (0.70–9.16)	
sian	0.88 (0.11–6.82)	
Other	1.09 (0.01–10.3)	
Hepatitis C Antibody Positive	5.97 (1.47–24.24)	0.01
Current Smoker	0.96 (0.34–2.76)	0.94
Diabetes Mellitus	0.47 (0.11–2.05)	0.31
Coronary Artery Disease	2.48 (0.89–6.90)	0.08
Peripheral Vascular Disease	0.83 (0.10–7.11)	0.87
Cerebrovascular Disease	2.00 (0.26–15.13)	0.50
Previous Diagnosis of Cancer	3.46 (1.06–11.25)	0.04
Chronic Lung Disease	3.79 (1.17–12.27)	0.03
First KRT modality		
HD	Ref	0.09
PD	0.78 (0.31–1.98)	
Pre-emptive transplant	0.13 (0.02–1.07)	
Late referral to dialysis	0.21 (0.05–0.97)	0.05
Transplant era 2007–16	1.97 (0.83–4.66)	0.12

*ATSI* Aboriginal and Torres Strait Islander, *MPI* Maori and Pacific Islander, *KRT* Kidney replacement therapy, *HD* Hemodialysis, *PD* Peritoneal dialysis

### Kidney allograft survival

**Fig. 4:**
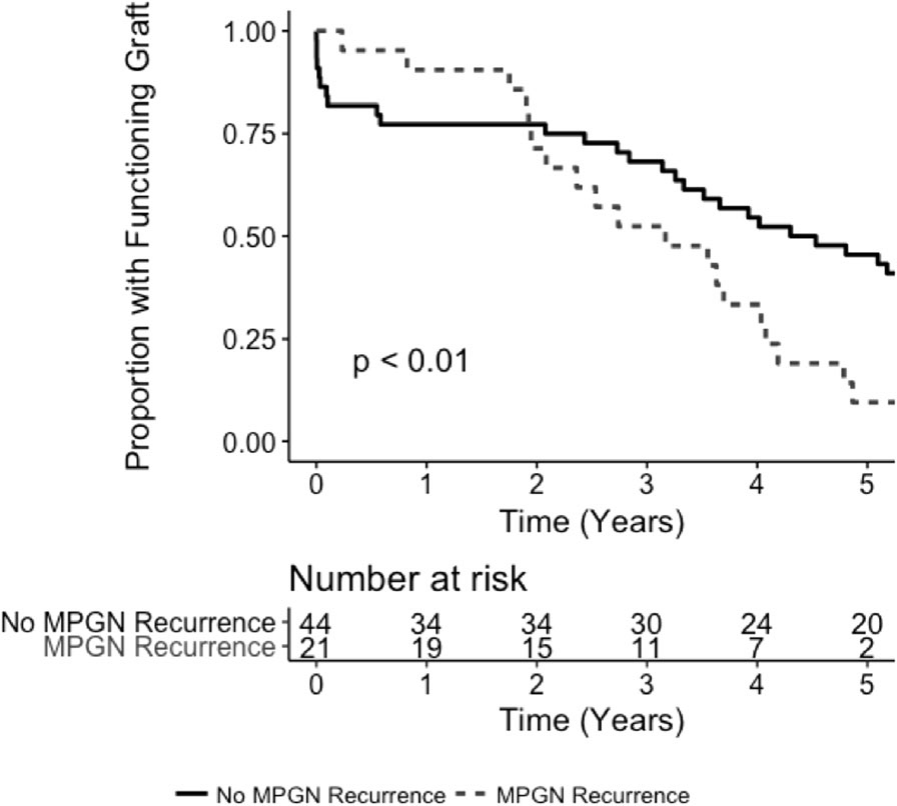
Kaplan Meier Survival analysis of kidney allograft survival in individuals with MPGN vs. matched controls with all other GN who received a kidney transplant in Australia and New Zealand between 1996 and 2016

During the study, kidney allograft loss occurred in 65 (34%) patients with ESKD due to MPGN and 934 (31%) patients with ESKD due to all other GN (*p* = 0.34). The most common cause of allograft loss for both groups was chronic allograft nephropathy (35% for MPGN and 53% for all other GN). Five-year allograft survival was 70% for patients with MGPN and 81% for patients with all other GN (*p* = 0.02; Fig. 4). Using multivariable Cox regression, allograft failure occurred significantly more frequently in patients with MPGN than in those with all other GN (HR 1.46, 95% CI 1.02–2.09, *p* < 0.01; Additional file 1: Table S14).

Five-year death censored allograft survival was also lower for patients with MPGN compared to patients with all other GN (74% vs. 87%, respectively, *p* < 0.01). Similar results were observed with Cox regression (HR 1.91, 95% CI 1.24–2.94, *p* < 0.01; Additional file 1: Table S15) and competing risks analysis (SHR 1.76, 95% CI 1.23–2.54, *p* = 0.01; Additional file 1: Table S16).

## Discussion

This is the first study to have comprehensively described the dialysis and transplant outcomes of patients with ESKD due to MPGN in the contemporary setting. While patient survival rates on dialysis and following transplantation were comparable to those of patients with other types of glomerulonephritis, higher rates of MPGN recurrence were associated with poorer kidney allograft survival rates.

In keeping with the findings of an earlier US study [1], patients with MPGN had similar survival rates following kidney transplantation to those with other forms of glomerulonephritis. These observations were extended in the present study by demonstrating similar patient survival rates between the two groups in the setting of chronic dialysis. The fact that MPGN most commonly presents with kidney-limited symptoms and rarely affects other major organs [8] may have helped to mitigate the adverse impact of a diagnosis of MPGN on patient survival.

On the other hand, kidney allograft survival and death censored allograft survival were substantially poorer in patients with MPGN compared to patients with any other form of glomerulonephritis. This is in keeping with previous findings from the United Network for Organ Sharing (UNOS) database from 1987 to 2007, which reported significantly lower 10 year death-censored allograft survival rates in patients with MPGN compared with other forms of glomerulonephritis (56.2% vs 65.2%, *p* < 0.001) [1]. Disease recurrence was the most common reported cause of allograft failure in patients with MPGN (14.5%) and was significantly more common than in patients with other glomerulonephritis (6.6%, *p* < 0.001). In contrast, the corresponding figures in the present study were 32 and 5%, respectively, which may have reflected improved recognition of disease recurrence as a common cause of early allograft loss in patients with MPGN.

The pathogenesis of MPGN is complex and the reason why patients with MPGN have higher rates of disease recurrence remains unclear [8]. The recent advances in the classification of MPGN have raised the possibility that the increased risk of recurrence may be due to patients with C3GN [17]. The cause of C3GN is still being investigated but several genetic mutations (eg. C3, CFH, CD46, CFB) have been associated with its onset [18]. It has been postulated that the higher rates of disease relapse in transplant recipients with MPGN could be associated with specific gene mutations in patients with C3GN [17]. Unfortunately, due to the historical nature of our study, the current classification system used for MPGN was not available and it was not possible to assess the effect of the different mechanisms of immune injury in MPGN on allograft survival or rates of disease recurrence.

Low complement levels, the presence of monoclonal bands, living related kidney donors and pre-emptive transplantation have all been identified as risk factors for disease recurrence [7]. While complement levels and monoclonal protein bands could not be assessed in this study, there was no difference in the risk of MPGN recurrence in living related donors or pre-emptive donors compared to other donor sources in this study. The association between living related donors, pre-emptive donors and MPGN recurrence was previously described in a single center study with a small patient cohort [7]. It may be that this earlier finding represents a specific regional variation that is not more broadly generalizable.

MPGN was also found to be a rare cause of ESKD in the present study, affecting 0.8% of ESKD patients. Similar findings have been reported previously [4], including in the United States Renal Data System (USRDS) registry [1], which identified that MPGN accounted for only 0.33% of patients with ESKD. The incidence of MPGN also decreased during the study duration, in line with internationally observed trends [1, 4]. Hepatitis C infection has previously been identified as a major cause of MPGN [8]. Although the incidence of hepatitis C in Australia has been falling since 1999 [19], a similar fall in hepatitis C prevalence was not observed in the present study. Hepatitis C treatment was not accessible to patients with CKD and ESKD during the course of the study, although it has become freely available in Australia since 2018 [20]. As this treatment is increasingly utilized in patients with chronic kidney disease, it is likely that rates of MPGN will continue to fall.

Maori and Pacific Islanders were found to be more likely to develop MPGN compared to other ethnicities, which is consistent with previous reports [21, 22]. Potential explanations for this observation include higher rates of socio-economic disadvantage, infectious disease and delayed diagnoses, as well as reduced access to healthcare compared to other ethnic groups [22, 23].

Findings from the current study are strengthened by the large cohort of patients and represent the most comprehensive description of outcomes in MPGN patients with ESKD in the contemporary era. All patients in the MPGN cohort were biopsy confirmed, thereby enhancing study validity. The limitations of the study are similar to those of other retrospective cohort studies. Misclassification or under-reporting of registry data was possible and inspection of patient records was not feasible. The registry did not contain important information that could affect the outcomes of patients with MPGN. These include the cause of MPGN, the patient course prior to commencing KRT (eg. a rapidly progressive course, nephrotic syndrome, time from diagnosis to commencing KRT) and the duration and type of treatment provided prior to KRT. Additionally, immunosuppressive therapy received at baseline following transplantation or after diagnosis of disease recurrence in the allograft was not available and its impact on patient and allograft survival could not be assessed. While hepatitis C status was available, no further information regarding the presence of liver disease or type 2 cryoglobulinaemia was recorded in the registry and it was not possible to assess the impact of liver disease or systemic manifestations of hepatitis C on MPGN rates, disease recurrence or patient survival. The presence of monoclonal protein bands and low complement levels has previously been identified as being associated with MPGN recurrence [7]. These were also not available from the registry data provided. The absence of this data limits the inferences that can be drawn from this study.

MPGN is a rare condition and there is a risk that unmeasured bias may have arisen from matching two groups that significantly differ in size. The prevalence of patients with MPGN within the cohort was much lower (approximately 1%) compared to patients with other GN (22%). This difference in prevalence increases the risk of bias being introduced through propensity score matching. This risk was mitigated through utilizing nearest neighbor matching with a small caliper width [24] which allowed a mean standardized differences of less than 0.1 for all covariates used. While these matching techniques minimize as much as possible the risk of bias being introduced through matching [24], it cannot completely exclude the possibility of unmeasured bias in the matched cohorts.

## Conclusion

Patients with MPGN had similar mortality on dialysis and following kidney transplantation compared to patients with any other glomerulonephritis, but had significantly higher rates of allograft and death censored allograft loss that were in large part due to higher rates of disease recurrence. Overall, this study highlights the necessity for further research to understand the reason for increased rates of disease recurrence among patients with MPGN, and identifies the unique challenges faced by patients with this rare cause of ESKD.

## Supplementary information

**Additional file 1.** Supplementary Tables and Figures of Patients with MPGN compared to Patients with any other GN. 18 tables containing descriptive data of patients with MPGN and other GN data referenced in the text as Tables S1–S18 and 4 figures containing additional analysis of patients with MPGN and other GN referenced in the text as Figures S1-S4.

## Data Availability

The data that supports the findings of this study are available from ANZDATA but restrictions apply to the availability of this data, which were used under license for the current study, and so are not publicly available. Data are however available from the authors upon reasonable request and with permission of ANZDATA.

## Abbreviations

ANZDATA: Australian and New Zealand Dialysis and Transplantation Registry
ESKD: End stage kidney disease
GN: Glomerulonephritis
HR: Hazard ratio
KRT: Kidney replacement therapy
MPGN: Membranoproliferative glomerulonephritis
SHR: Sub-hazard ratio
USRDS: United States Renal Data System
