# Supplementary material for: Long-term outcomes of patients with end-stage kidney disease due to membranoproliferative glomerulonephritis: an ANZDATA registry study
Source: BMC Nephrol. 2019 Nov 21;20:417. doi: 10.1186/s12882-019-1605-6 (PMC6868684; doi:10.1186/s12882-019-1605-6)

**Supplementary Tables and Figures of Patients with MPGN compared to patients with any other GN**

**Table S1.** Types of Glomerulonephritis in dialysis patients with any other GN

| **Glomerulonephritis** | **N (%)** |
| --- | --- |
| IgA Nephropathy | 3196 (27%) |
| Presumed GN | 2853 (24%) |
| Primary focal segmental glomerulosclerosis | 2351 (20%) |
| Membranous glomerulonephritis | 617 (5%) |
| ANCA vasculitis | 615 (5%) |
| Lupus nephritis | 558 (5%) |
| GN not otherwise specified | 428 (4%) |
| Advanced glomerulonephritis (end-stage) | 379 (3%) |
| Rapidly progressive GN not otherwise specified | 312 (3%) |
| Anti-GBM disease | 302 (3%) |
| Familial GN | 273 (2%) |
| Scleroderma | 145 (1%) |

**Table S2.** Ethnicity of dialysis patients with MPGN over dialysis eras.

| **Ethnicity** | **1996-06 (n)** | **2007-16** |
| --- | --- | --- |
| Caucasian | 187 (68%) | 124 (68%) |
| ATSI | 31 (11%) | 9 (5%) |
| MPI | 39 (14%) | 31 (17%) |
| Asian | 14 (5%) | 12 (7%) |
| Other | 3 (1%) | 6 (3%) |

ATSI, Aboriginal and Torres Strait Islander; MPI, Maori and Pacific Islander

**Table S3.** Cause of Death for Patients on Dialysis.

| **Cause** | **MPGN** | **Other Glomerulonephritis** |
| --- | --- | --- |
| Cardiac | 72 (33%) | 1681 (32%) |
| Withdrawal | 48 (22%) | 1011 (19%) |
| Infection | 42 (19%) | 1185 (23%) |
| Cancer | 20 (9%) | 437 (8%) |
| Vascular | 20 (9%) | 416 (8%) |
| Other | 18 (8%) | 504 (10%) |

**Table S4.** Cause of Death for Patients following Kidney Transplantation.

| **Cause of Death** | **MPGN** | **Other Glomerulonephritis** |
| --- | --- | --- |
| Infection | 12 (32%) | 163 (21%) |
| Cardiac | 7 (19%) | 177 (23%) |
| Cancer | 7 (19%) | 198 (26%) |
| Withdrawal | 4 (11%) | 66 (9%) |
| Vascular | 4 (11%) | 57 (7%) |
| Other | 3 (8%) | 100 (13%) |

Patients who underwent kidney transplant and had allograft failure and returned to dialysis were included in this cohort

**Table S5**. Cox Proportional Hazard Analysis for Kidney Recovery in Patients with MPGN vs other glomerulonephritis.

| **Variable** | **HR (95% CI)** | **P** |
| --- | --- | --- |
| MPGN | 1.36 (0.79-2.35) | 0.26 |
| Age | 1.01 (1.00-1.02) | 0.02 |
| Male | 0.67 (0.53-0.85) | <0.01 |
| Ethnicity |  |  |
| Caucasian | Ref | <0.01 |
| ATSI | 1.04 (0.65-1.66) |  |
| MPI | 0.71 (0.45-1.13) |  |
| Asian | 0.37 (0.21-0.66) |  |
| Other | 0.68 (0.28-1.67) |  |
| Hepatitis C Antibody Positive | 1.43 (0.74-2.76) | 0.29 |
| Current Smoker | 1.35 (0.99-1.83) | 0.06 |
| Diabetes Mellitus | 0.95 (0.68-1.32) | 0.75 |
| Coronary Artery Disease | 0.87 (0.64-1.2) | 0.41 |
| Peripheral Vascular Disease | 1.09 (0.72-1.66) | 0.68 |
| Cerebrovascular Disease | 1.23 (0.81-1.87) | 0.32 |
| Previous Diagnosis of Cancer | 0.68 (0.42-1.09) | 0.11 |
| Chronic Lung Disease | 1.5 (1.11-2.03) | <0.01 |
| First KRT modality |  |  |
| HD | Ref | 0.23 |
| PD | 0.83 (0.62-1.12) |  |
| Late referral to dialysis | 3.43 (2.71-4.34) | <0.01 |
| Dialysis era 2007-16 | 2.04 (1.61-2.59) | <0.01 |

ATSI, Aboriginal and Torre Strait Islander; MPI, Maori and Pacific Islander; Patients with other GD was the reference group for patients with MPGN, Caucasian ethnicity was used as the reference group for all other ethnicities; HD was the reference group for first dialysis modality

**Table S6.** Disease Relapse in patients of MPGN by Hepatitis C Antibody Status.

|  | **No disease relapse** | **Disease relapse** |
| --- | --- | --- |
| Hepatitis C Ab Positive | 14 | 1 |
| Hepatitis C Ab Negative | 159 | 34 |

ATSI, Aboriginal and Torre Strait Islander; MPI, Maori and Pacific Islander

**Table S7.** Disease relapse in patients with MPGN by patient ethnicity.

|  | **No Disease Relapse** | **Disease Relapse** |
| --- | --- | --- |
| Caucasian | 131 | 29 |
| ATSI | 7 | 1 |
| MPI | 19 | 4 |
| Asian | 13 | 1 |
| Other | 3 | 0 |

p=0.76

ATSI, Aboriginal and Torre Strait Islander; MPI, Maori and Pacific Islander

**Table S8.** Disease recurrence in patients with MPGN by donor source.

|  | **No disease recurrence** | **Disease recurrence** |
| --- | --- | --- |
| Deceased donor | 97 (81%) | 23 (19%) |
| Living related | 40 (83%) | 8 (17%) |
| Unrelated living donor | 19 (86%) | 3 (14%) |

**Table S9.** Causes of allograft loss in patients with MPGN vs other glomerulonephritis.

|  | **Other Glomerulonephritis** | **MPGN** |
| --- | --- | --- |
| Acute rejection | 58 (6%) | 1 (2%) |
| Chronic allograft nephropathy | 497 (53%) | 23 (35%) |
| De Novo GN | 68 (7%) | 5 (8%) |
| Drug therapy | 82 (9%) | 2 (3%) |
| Other | 75 (8%) | 5 (8%) |
| GN Recurrence | 58 (6%) | 21 (32%) |
| Technical loss | 19 (2%) | 1 (2%) |
| Vascular complication | 77 (8%) | 7 (11%) |

**Table S10.** Standardised mean differences for propensity score matching for the dialysis cohort.

| **Variable** | **Standardised Mean Differences** |
| --- | --- |
| Caucasian | 0.00 |
| ATSI | -0.09 |
| MPI | 0.08 |
| Asian | -0.03 |
| Other | 0.03 |
| Age | -0.07 |
| Male | -0.09 |
| Hepatitis C Antibody | 0.03 |
| Smoking status | 0.03 |
| Diabetes Mellitus | 0.04 |
| Coronary Artery Disease | 0.06 |
| Peripheral Vascular Disease | 0.08 |
| Cerebrovascular disease | 0.07 |
| History of cancer | 0.03 |
| Chronic Lung Disease | 0.02 |
| KRT type | -0.01 |
| Late referral | 0.03 |
| Dialysis Era | 0.02 |

**Table S11.** Standardised mean differences for propensity score matching for the kidney transplant cohort.

| **Variable** | **Standardised Mean Differences** |
| --- | --- |
| Caucasian | -0.05 |
| ATSI | 0.09 |
| MPI | 0.02 |
| Asian | 0.02 |
| Other | -0.04 |
| Age | 0.09 |
| Male | 0 |
| Hepatitis C Antibody | -0.02 |
| Smoking status | -0.04 |
| Diabetes Mellitus | -0.08 |
| Coronary Artery Disease | 0.05 |
| Peripheral Vascular Disease | 0.04 |
| Cerebrovascular disease | 0.04 |
| History of cancer | -0.06 |
| Chronic Lung Disease | 0.036 |
| First KRT type | -0.02 |
| Late referral | -0.01 |
| Transplant Era | -0.01 |

**Table S12.** Competing risk regression hazard ratios for dialysis survival.

| **Variable** | **SHR (95% CI)** | **P** |
| --- | --- | --- |
| MPGN | 0.98 (0.83-1.17) | 0.86 |
| Age | 1.05 (1.05-1.06) | <0.01 |
| Male | 0.83 (0.69-0.99) | 0.09 |
| Non-Smoker | 0.68 (0.54-0.85) | <0.01 |
| No chronic lung disease | 0.80 (0.63-1.03) | 0.14 |
| No coronary artery disease | 0.68 (0.55-0.84) | <0.01 |
| No peripheral vascular disease | 0.68 (0.51-0.92) | 0.03 |
| No cerebrovascular disease | 0.54 (0.40-0.74) | <0.01 |
| No diabetes | 0.87 (0.70-1.07) | 0.26 |
| Previous diagnosis of Cancer | 1.19 (0.92-1.53) | 0.27 |
| No late referral to KRT | 0.88 (0.72-1.07) | 0.27 |
| Hepatitis C Antibody negative | 0.67 (0.50-0.89) | 0.02 |
| ATSI | 2.47 (1.84-3.33) | <0.01 |
| MPI | 2.24 (1.71-2.93) | <0.01 |
| Asian | 0.62 (0.39-0.99) | 0.09 |
| Other | 0.29 (0.11-0.72) | 0.03 |
| First dialysis modality PD | 1.20 (0.99-1.45) | 0.12 |
| Dialysis era 07-16 | 0.64 (0.53-0.79) | <0.01 |

**Table S13.** Competing risk regression for transplant patient survival.

|  | **SHR (95% CI)** | **P** |
| --- | --- | --- |
| MPGN | 0.61 (0.34-1.11) | 0.17 |
| Age | 1.07 (1.04-1.10) | <0.01 |
| Male | 0.83 (0.45-1.54) | 0.63 |
| Current Smoker | 1.12 (0.42-2.95) | 0.85 |
| No chronic lung disease | 0.31 (0.09-1.03) | 0.11 |
| No coronary artery disease | 0.43 (0.16-1.20) | 0.18 |
| No peripheral vascular disease | 1.07 (0.11-10.10) | 0.96 |
| No cerebrovascular disease | 0.76 (0.11-5.43) | 0.82 |
| No diabetes | 0.93 (0.28-3.07) | 0.92 |
| Previous diagnosis cancer | 3.67 (1.56-8.66) | 0.01 |
| No late referral | 3.74 (1.00-13.79) | 0.10 |
| Negative hepatitis C antibody | 0.28 (0.08-0.99) | 0.10 |
| First dialysis modality PD | 0.68 (0.30-1.52) | 0.43 |
| First dialysis modality Transplant | 0.20 (0.05-0.76) | 0.05 |
| Transplant era 2007-16 | 1.35 (0.71-2.54) | 0.44 |
| ATSI | 14.40 (3.38-61.60) | <0.01 |
| MPI | 1.86 (0.65-5.31) | 0.33 |
| Asian | 0.70 (0.12-4.00) | 0.73 |
| Other | 0.05 (0.01-0.11) | <0.01 |

**Table S14.** Cox proportional hazards for kidney allograft survival.

|  | **HR (95% CI)** | **P** |
| --- | --- | --- |
| MPGN | 1.46 (1.02-2.09) | 0.04 |
| Age | 1.00 (0.99-1.02) | 0.83 |
| Male | 0.96 (0.66-1.39) | 0.82 |
| Ethnicity |  |  |
| Caucasian | Ref | 0.04 |
| ATSI | 2.24 (0.86-5.80) |  |
| MPI | 2.02 (1.22-3.33) |  |
| Asian | 0.87 (0.37-2.04) |  |
| Other | 0.72 (0.10-5.28) |  |
| Hepatitis C Antibody Positive | 1.48 (0.69-3.17) | 0.32 |
| Current Smoker | 1.30 (0.80-2.13) | 0.29 |
| Diabetes Mellitus | 1.23 (0.56-2.72) | 0.60 |
| Coronary Artery Disease | 1.29 (0.60-2.81) | 0.52 |
| Peripheral Vascular Disease | 1.13 (0.30-4.21) | 0.86 |
| Cerebrovascular Disease | 1.3 (0.43-3.90) | 0.64 |
| Previous Diagnosis of Cancer | 1.22 (0.48-3.09) | 0.68 |
| Chronic Lung Disease | 1.45 (0.72-2.91) | 0.29 |
| First KRT modality |  |  |
| HD | Ref | 0.23 |
| PD | 0.81 (0.52-1.28) |  |
| Pre-emptive transplant | 0.53 (0.24-1.16) |  |
| Late referral to dialysis | 0.73 (0.43-1.25) | 0.25 |
| Transplant era 2007-16 | 1.04 (0.69-1.57) | 0.86 |

**Table S15.** Cox proportional hazards for death censored allograft survival.

|  | **HR (95% CI)** | **P** |
| --- | --- | --- |
| MPGN | 1.91 (1.24-2.94) | 0.003 |
| Age | 0.98 (0.97-1.00) | 0.026 |
| Male | 0.99 (0.64-1.52) | 0.954 |
| Ethnicity |  |  |
| Caucasian | Ref | 0.17 |
| ATSI | 1.09 (0.30-3.94) |  |
| MPI | 1.88 (1.08-3.27) |  |
| Asian | 0.98 (0.38-2.50) |  |
| Other | 0.68 (0.09-5.09) |  |
| Hepatitis C Antibody Positive | 1.03 (0.40-2.68) | 0.951 |
| Current Smoker | 1.53 (0.86-2.70) | 0.149 |
| Diabetes Mellitus | 2.08 (0.75-5.73) | 0.159 |
| Coronary Artery Disease | 0.41 (0.09-1.82) | 0.244 |
| Peripheral Vascular Disease | 0.55 (0.07-4.49) | 0.576 |
| Cerebrovascular Disease | 1.09 (0.26-4.64) | 0.905 |
| Previous Diagnosis of Cancer | 0.38 (0.05-2.80) | 0.339 |
| Chronic Lung Disease | 1.14 (0.46-2.86) | 0.774 |
| First KRT modality |  |  |
| HD | Ref | 0.57 |
| PD | 0.83 (0.49-1.41) |  |
| Pre-emptive transplant | 0.68 (0.29-1.60) |  |
| Late referral to dialysis | 0.89 (0.50-1.59) | 0.696 |
| Transplant era 2007-16 | 0.78 (0.48-1.27) | 0.312 |

**Table S16.** Competing risk regression for death censored allograft survival.

|  | **SHR (95% CI)** | **P** |
| --- | --- | --- |
| MPGN | 1.76 (1.23-2.54) | 0.01 |
| Age | 0.98 (0.96-0.99) | <0.01 |
| Male | 1.03 (0.73-1.45) | 0.89 |
| Current smoker | 1.63 (1.03-2.58) | 0.08 |
| No chronic lung disease | 0.75 (0.44-1.27) | 0.37 |
| No coronary artery disease | 1.67 (0.61-4.57) | 0.40 |
| No peripheral vascular disease | 0.69 (0.30-1.58) | 0.46 |
| No cerebrovascular disease | 0.97 (0.31-3.04) | 0.96 |
| No diabetes | 0.72 (0.31-1.66) | 0.51 |
| Previous diagnosis cancer | 0.33 (0.05-1.95) | 0.30 |
| No late referral | 1.09 (0.69-1.71) | 0.76 |
| Negative Hepatitis C antibody | 1.08 (0.50-2.36) | 0.86 |
| ATSI | 1.60 (0.62-4.09) | 0.41 |
| MPI | 1.87 (1.16-3.03) | 0.03 |
| Asian | 1.32 (0.68-2.54) | 0.49 |
| Other | 0.65 (0.10-3.99) | 0.69 |
| First modality PD | 0.88 (0.57-1.37) | 0.65 |
| First modality Transplant | 0.74 (0.37-1.48) | 0.48 |
| Transplant era 07-16 | 0.81 (0.54-1.20) | 0.38 |

**Table S17.** Number of patients surviving with MPGN vs. other GN on dialysis in matched cohort over first five years

| Year | 1 | 2 | 3 | 4 | 5 |
| --- | --- | --- | --- | --- | --- |
| MPGN | 349 | 281 | 209 | 170 | 129 |
| Other GN | 364 | 287 | 220 | 180 | 124 |

**Table S18.** Number of patients surviving with MPGN vs. other GN following transplant in matched cohort over first five years

| Year | 1 | 2 | 3 | 4 | 5 |
| --- | --- | --- | --- | --- | --- |
| MPGN | 169 | 149 | 137 | 122 | 109 |
| Other GN | 167 | 156 | 141 | 127 | 114 |

**Figure S1.** Flow Diagram of Patient Cohort Selection.

56481 patients receiving kidney replacement therapy from 1996-2016

43365 patients receiving kidney replacement therapy from 1996-2016 with any other cause of ESKD or incomplete patient covariate data excluded.

13116 patients receiving kidney replacement therapy with MPGN or any other glomerulonephritis as cause of ESKD included for analysis

**Figure S2.** Cumulative incidence of time to first kidney transplant for patients with MPGN vs. other glomerulonephritis.


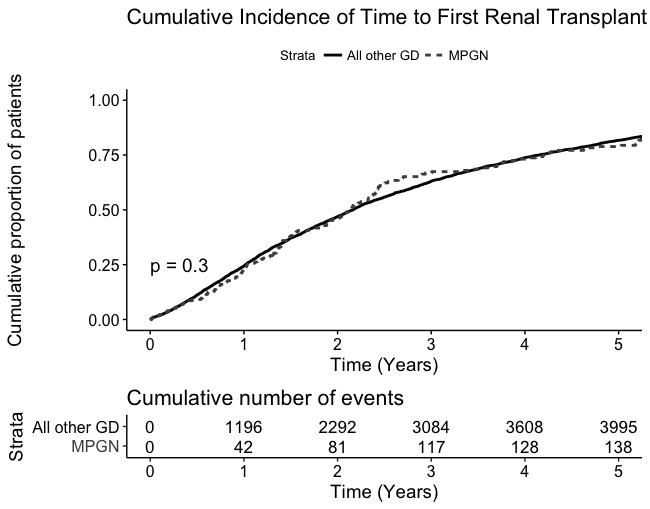


**Figure S3.** Box Plot of propensity scores for dialysis matched cohorts.


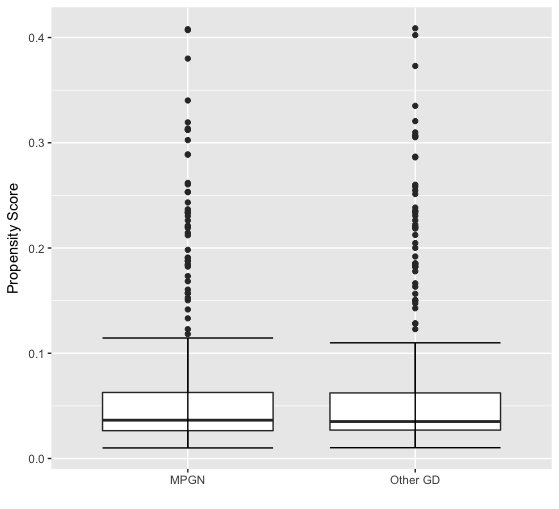


**Figure S4.** Box Plot of propensity scores for transplant matched cohorts.


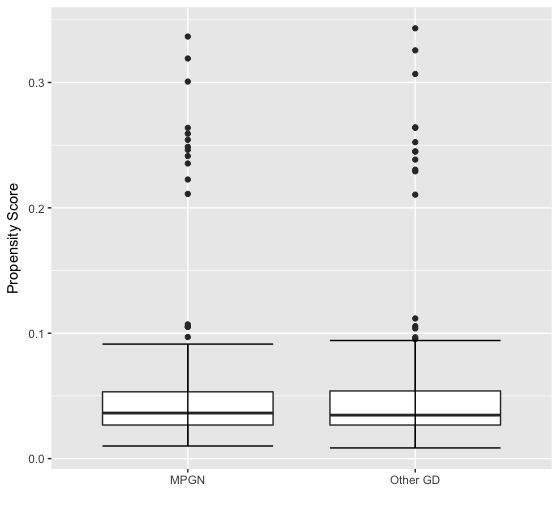

Supplement: Supplementary file 1 — Additional file 1. Supplementary Tables and Figures of Patients with MPGN compared to Patients with any other GN. 18 tables containing descriptive data of patients with MPGN and other GN data referenced in the text as Tables S1–S18 and 4 figures containing additional analysis of patients with MPGN and other GN referenced in the text as Figures S1-S4. [file 12882_2019_1605_MOESM1_ESM.docx]
